# Modeling Abundance of *Culicoides stellifer*, a Candidate *Orbivirus* Vector, Indicates Nonrandom Hemorrhagic Disease Risk for White-Tailed Deer (*Odocoileus virginianus*)

**DOI:** 10.3390/v13071328

**Published:** 2021-07-09

**Authors:** Emily T. N. Dinh, Juan Pablo Gomez, Jeremy P. Orange, Max A. Morris, Katherine A. Sayler, Bethany L. McGregor, Erik M. Blosser, Nathan D. Burkett-Cadena, Samantha M. Wisely, Jason K. Blackburn

**Affiliations:** 1 Spatial Epidemiology & Ecology Research Laboratory, Department of Geography, University of Florida, 3141 Turlington Hall, Gainesville, FL 32611, USA; dinhe@michigan.gov (E.T.N.D.); jugomez@ufl.edu (J.P.G.); jporange2@ufl.edu (J.P.O.); maxmorris1@ufl.edu (M.A.M.); 2 Emerging Pathogens Institute, University of Florida, 2055 Mowry Road, Gainesville, FL 32611, USA; 3 Department of Wildlife Ecology and Conservation, 110 Newins-Ziegler Hall, Gainesville, FL 32611, USA; ksayler1@gmail.com (K.A.S.); wisely@ufl.edu (S.M.W.); 4 Florida Medical Entomology Laboratory, University of Florida, 200 9th St SE, Vero Beach, FL 32962, USA; bmcgreg1@gmail.com (B.L.M.); emblosser@ucdavis.edu (E.M.B.); nburkettcadena@ufl.edu (N.D.B.-C.)

**Keywords:** epizootic hemorrhagic disease virus, occupancy model, *Culicoides* *stellifer*, disease transmission, vector-borne disease, spatial model, GIS

## Abstract

(1) Background: Hemorrhagic diseases in white-tailed deer (*Odocoileus* *virginianus*) are caused by orbiviruses and have significant economic impact on the deer ranching industry in the United States. *Culicoides* *stellifer* is a suspected vector of epizootic hemorrhagic disease virus (EHDV), with recent field evidence from Florida, but its natural history is poorly understood. Studying the distribution and abundance of *C. stellifer* across the landscape can inform our knowledge of how virus transmission can occur locally. We may then target vector management strategies in areas where viral transmission can occur. (2) Methods: Here, we used an occupancy modeling approach to estimate abundance of adult *C. stellifer* females at various physiological states to determine habitat preferences. We then mapped midge abundance during the orbiviral disease transmission period (May–October) in Florida. (3) Results: We found that overall, midge abundance was positively associated with sites in closer proximity to large-animal feeders. Additionally, midges generally preferred mixed bottomland hardwood and agricultural/sand/water habitats. Female *C. stellifer* with different physiological states preferred different habitats. (4) Conclusions: The differences in habitat preferences between midges across states indicate that disease risk for deer is heterogeneous across this landscape. This can inform how effective vector management strategies should be implemented.

## 1. Introduction

Captive breeding of native and exotic cervid species (Artiodactyla: Cervidae) for commercial purposes is one of the fastest-growing industries in the rural United States. As of 2017, this industry generates $8 billion for the US economy and supports nearly 60,000 jobs, most of which are in rural areas [1]. Most of the industry raises white-tailed deer (WTD; *Odocoileus virginianus*), which is also a valuable wild game species throughout the US. Infectious diseases caused by orbiviruses (Reoviridae), mainly epizootic hemorrhagic disease virus (EHDV) and bluetongue virus (BTV), can cause serious morbidity and death. The diseases caused by these two viruses are collectively called hemorrhagic disease (HD). There are no treatments available for WTD affected by HD, so outbreaks of either can result in significant economic losses.

Both viruses are transmitted by adult female *Culicoides* biting midges (Diptera: Ceratopogonidae). Our current understanding of the geographic distributions of EHDV and BTV in North America is partly based on the distribution of *Culicoides sonorensis*, the principal vector of BTV and the only confirmed vector of EHDV [2,3]. However, in endemic regions of the southeastern US such as Florida, *C. sonorensis* has been rarely collected, so it is likely that other midge species are responsible for transmitting EHDV and BTV in this area [3]. Unfortunately, relatively little is known about the ecology and life history of many *Culicoides* species occurring in Florida [4,5]. Consequently, there are few methods that vector biologists and livestock producers can utilize to respond to disease outbreaks or severe nuisance problems caused by *Culicoides* [5]. While control may be difficult, if not untenable [6,7], better understanding vector ecology may improve our estimates of HD risk.

One way to better understand HD dynamics in Florida is to study the distribution and abundance of the vectors across the landscape. *Culicoides stellifer* is a candidate vector of EHDV and BTV in Florida, due to its propensity for feeding on WTD [8] (McGregor et al., 2018), being the most abundant species trapped on deer farms during the HD transmission season in previous Florida studies [3,8], and being naturally infected with EHDV [8]. Modeling spatially replicated counts of *C. stellifer* can uncover what factors correlate to abundance patterns in space and time and, along with information on where vertebrate hosts are likely to occur, where on a landscape virus transmission can occur. Relating site-specific characteristics to habitat selection by insect vectors with poorly known natural histories may help incriminate candidate vectors [9,10] and guide efforts to reduce WTD morbidity and mortality from HD via vector control [11].

Identifying where and when transient populations of potentially infectious vectors occur is paramount for modeling HD risk and could inform vector management actions that reduce a local midge population, even if to better inform the challenges [7]. Since evidence of vertical orbiviral transmission amongst both invertebrate and vertebrate hosts in North America is currently lacking [3,12], we assume that EHDV and BTV have enzootic cycles involving mammal hosts and adult female *Culicoides*. Thus, vector ecology studies should target vector sampling at the physiological state(s) most important for pathogen transmission [13]. To determine habitat preferences in this study, we modeled the abundance of adult *C. stellifer* females at each physiological status (nulliparous, parous, gravid, and bloodfed). Of these statuses, parous midges (females seeking blood meal hosts after completing a gonotrophic cycle) are those that transmit EHDV and BTV because they may have acquired virus from a previous blood meal on an infected vertebrate host, laid their first clutch of eggs, and then pursued another blood meal from a susceptible vertebrate host to which they may transfer viruses. Gravid midges would be of next highest importance because they have taken a blood meal, will add to the population of midges by laying eggs, then become parous and possibly transmit viruses. Bloodfed midges would be of lower concern because it is difficult to determine whether they have previously acquired a blood meal and laid eggs. A bloodfed midge may have gotten infected with virus from a prior blood meal. Nulliparous midges would be of lowest priority as they have not yet taken a blood meal and should not have acquired pathogens [9]. These cohorts likely have different biological needs and thus should have unique geographic distributions as they seek environments suitable to their specific needs.

Here, we investigated how site-specific *C. stellifer* abundance relates to environmental characteristics and changes over serial sampling events at fixed sites. Our specific questions for this research were (1) do *C. stellifer* of various physiological statuses prefer different habitats? and (2) if so, what are the characteristics of the habitat(s) preferred by *C. stellifer* of each physiological status? Additionally, we aimed to predict and map where populations of *C. stellifer* would likely occur during the HD transmission season. We hypothesized that *C. stellifer* at each physiological state would choose different environments to suit their biological needs. For example, gravid midges may likely occur near appropriate oviposition habitats such as riversides and pond edges while parous midges may select areas highly used by vertebrate hosts [4].

## 2. Materials and Methods

### 2.1. Study Area

**Figure 1:**
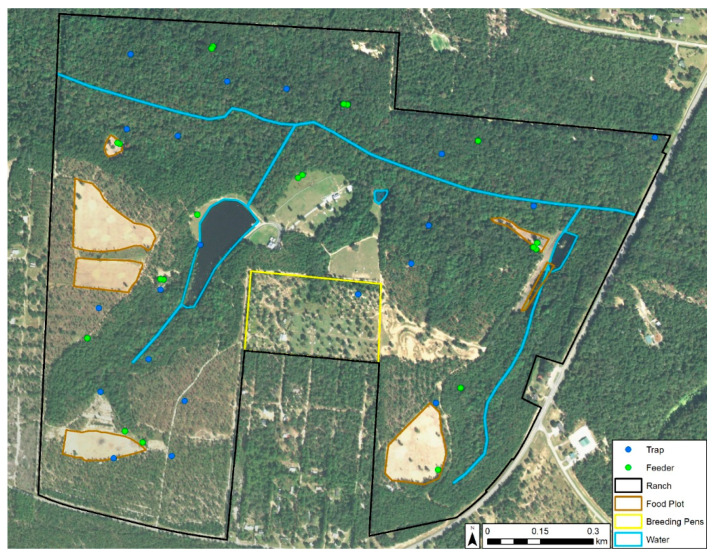
Features and insect trap locations (blue dots) on the study ranch in Gadsden County, Florida. 2015 satellite imagery from USGS EarthExplorer. When two feeders (green dots) appear side by side, one is for seasonal corn.

This study was conducted within an approximately 200 ha privately owned, high-fenced deer ranch in Gadsden County, Florida. The primary management objective of the ranch was cervid propagation. During our study period, there were approximately 130–150 free-ranging WTD managed with food plots and 12 stationary supplementary protein feeders regularly filled by farm staff [14]. The ranch also had approximately 150 exotic cervids and bovids of 13 different species on its property [8], yielding an animal density of approximately 1.48 animals/ha. The property also had 11 high-fenced WTD breeding pens occupying ≃9.3 ha in total that were inaccessible to free-ranging animals (Figure 1). The dominant landscape on the property was hardwood hammock. Upland short leaf pine species such as loblolly pine (*Pinus taeda*) were a prominent feature on the property.

#### Entomological Sampling

The following collection method is fully detailed in McGregor et al. [8]. Briefly, we used the random point generator in ArcGIS Desktop 10.3.1 [15] to select 20 trap locations that represented all habitat types on the property and were spatially random (based on the average nearest neighbor statistic) to reduce spatial bias in modeling (Figure 1). At each site, we hung 1 miniature CDC light trap (Model 2836BQ, BioQuip, Rancho Dominguez, CA, USA) with a blacklight-emitting diode array (Model 2790 V390, BioQuip, Rancho Dominguez, CA, USA) from a 1.63 m-tall shepherd’s hook at a height of 1.37 m. Traps were powered via a 6 V-12 Ah gel-sealed battery (Model NP12-6, EnerSys, Reading, PA, USA) controlled by a timer to operate between 1 h prior to sunset and 1 h after sunrise. The trapping was conducted twice weekly between July 2015 and December 2016. Midges were identified to species utilizing the keys in Blanton and Wirth [4] and categorized as either nulliparous, parous, bloodfed, or gravid. For this research, we censored the collection data to only include *C. stellifer* females captured during the 2016 HD season (May–October), segregated the data by physiological status (total number of females, nulliparous, parous, gravid, and bloodfed), and grouped the data into one-week survey periods with two collections per week. We excluded observations that exceeded two collections in a week.

### 2.2. Environmental Data

**Figure 2:**
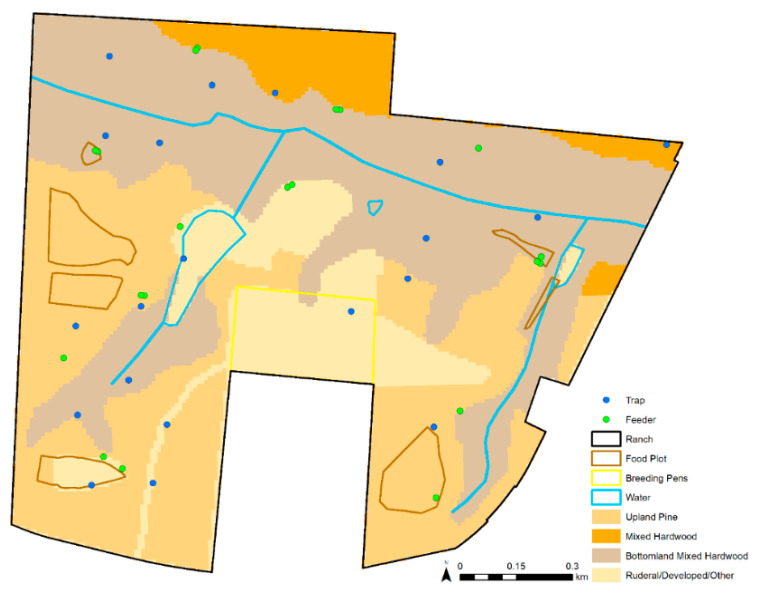
Habitat classes derived for this study on the study ranch.

We derived a total of six variables at trap locations to a 10 m raster for this study (Table 1). Three described landscape characteristics: distance to feeder, distance to water, and habitat type. We included the Euclidean distance from each trap site to the nearest feeder as a proxy for the availability of WTD, the most common blood meal host of female *C. stellifer* in the study property (McGregor et al. 2018). We also computed each trap location’s distance to the nearest permanent surface water body as a proxy for distance to potential oviposition sites because *C. stellifer* larvae are thought to occur in mud substrates [16,17]. We obtained data on permanent surface water sources that originated from the National Hydrography Dataset (NRCS) [18]. To test whether adult female *C. stellifer* at various physiological statuses preferred different land covers, we included habitat type as a model variable. The land cover data used in this research were derived from version 3.2 of the Cooperative Land Cover map created and managed by the Florida Fish and Wildlife Conservation Commission (FWC) and Florida Natural Areas Inventory (FNAI) [19]. There were initially too many vegetation types for our purposes, so we grouped similar vegetation communities to create one raster layer defining the habitat class at each pixel of the study ranch as upland pine, mixed hardwood pine, mixed bottomland hardwood, or rural/developed/other. These were inputted as unordered factor levels in our analyses. Definitions of the habitat classes are specified in Dinh et al. [20]. Habitat classes are mapped for the study ranch in Figure 2.

We estimated the probability of deer presence in the study environment by calculating utilization distributions (UDs) from collared animals we studied in Cauvin et al. [14] and Dinh [20]. UD can be defined as the probability density that an animal is found at a certain point in space without regard to environmental characteristics [21,22]. To obtain the UD, we first captured 15 ranched WTD, 1 fallow deer (*Dama dama*), and 1 Père David’s deer (*Elaphurus davidianus*) and outfitted them with GPS telemetry collars [20]. Capture and GPS outfitting protocols are fully described in Cauvin et al. (2020) and Dinh [20]. We included two non-native deer in UD construction because *C. stellifer* preferentially fed on fallow deer and occasionally on Pere David’s deer [8]. We censored the GPS location points to the HD transmission season and resampled all animals to a common interval of 60 min. Next, we pooled the GPS fixes and constructed a 10 m UD surface for each week in the HD season using the ‘adehabitatHR’ R package version 0.4.15 [23]. To explicitly account for space and time in our models of *C. stellifer* abundance, we included the standardized latitude-longitude coordinates of the centroid of each 10 m raster cell containing a trap site and survey period (week from beginning of season) as covariates.

### 2.3. Model Construction

Prior to model construction, we identified correlation between variables in R to avoid multicollinearity and model overfit. We computed Pearson correlation coefficients (r) to assess correlation between the numeric variables (distance to water, distance to feeder, and weekly UD value) and fitted ANOVA models to determine whether there were correlations between each pair of numerical and categorical variables. None of the Pearson’s r values exceeded |0.7| (Table 2) and none of the ANOVA tests indicated correlation between variables (Table 3). Finally, we extracted environmental covariate values at our 20 trap sites and standardized the continuous values before analyses to allow comparison of the impact of each variable on midge abundance [24,25,26]. Moreover, standardizing the site-level covariates helps stabilize the numerical optimization algorithm used during model construction [27].

For each physiological status of *C. stellifer*, we fitted repeated count models with all covariates as site-level covariates and the negative binomial prior mixing distribution using the ‘unmarked’ R package version 0.12-2 [27,28,29]. We selected the negative binomial distribution because insects are often not distributed randomly in space, and this distribution allows the density of animals to spatially vary [30]. Additionally, when we produced null models (described below) with various mixing distributions, the negative binomial models had the lowest AIC. Our abundance models were based on the N-mixture model described by Royle [10]:

(1) $$L\left(p,\theta |\left\{n_{it}\right\}\right)=\overset{R}{\underset{i=1}{\prod }}\left\{\overset{\infty }{\underset{N_{i}=max_{t}n_{it}}{\sum }}\left(\overset{T}{\underset{t=1}{\prod }}Bin(n_{it};N_{i},p)\right)f\left(N_{i};\theta \right)\right\}$$

where *p* is the detection (or capture) probability, *θ* is the mean of the prior mixing distribution on p, *R* the number of sampling locations, *n_it_* the number of individuals sampled at location *i* at time *t*, *N_i_* the number of individuals available for sampling, and *Bin*(*n_it_*; *N_i_*, *p*) is the binomial likelihood of the observed point count data. This model assumes that individuals are always available to collect during each sampling effort and lack of collection implies non-detectability. This is appropriate for our study because we studied a species so abundant that extraction of individuals during one sampling event was unlikely to affect the number of individuals available for detection in subsequent sampling events. Although our modeling approach can account for variation in detectability between surveys, we considered only site-level covariates to account for differences in *C. stellifer* abundance between trap locations and surveys. This is because time-specific survey-level covariates such as weather data at the study ranch were unavailable. If the model coefficient was negative for the distance to water or distance to feeder variables, a preference for that resource was indicated whereas a positive coefficient indicated avoidance. The opposite was true for land cover classes [31].

To explicitly test whether counts were spatially and/or temporally dependent, we first constructed four null models including all possible combinations of trap latitude-longitude locations and survey week as covariates. We selected the best null model of the four via AIC and model parsimony to serve as the basic model all the alternative models would be built upon.

We generated a list of models including all additive combinations of covariates. After completing model iteration for each physiological status of *C. stellifer*, we ranked models by ΔAIC and parsimony. We defined competing models as those with a ΔAIC < 2.0 because a difference greater than that will give strong evidence in favor of a model with the lowest AIC [32]. When models competed, we chose the one with the fewest parameters (i.e., the most parsimonious) as the best, final model [33]. Next, we predicted the weekly abundance of female *C. stellifer* of each physiological status across the study ranch with standardized covariates from their respective best models. Final maps of weekly predicted *C. stellifer* abundance during the 2016 HD risk period were produced in ArcGIS Desktop 10.3.1 [15]. Additionally, we saved the resulting rasters as JPEG files to create GIF animations of weekly predicted abundance across the study ranch during the HD season. Finally, we plotted actual weekly counts against predicted at each trap location to visualize model goodness of fit to the real data.

## 3. Results

### 3.1. Entomological Sampling

After filtering the entomological data for female *C. stellifer* trapped in the period May–October 2016, we caught a total of 21,533 specimens. Of these, 7515 were parous, 7308 were nulliparous, 6193 were gravid, and 517 were bloodfed.

#### Best Models for Each Physiological Stage of *C. stellifer*

The null model including survey period and standardized latitude-longitude locations of pixel centroids containing traps was the best null model for each physiological status and overall abundance (i.e., when physiological stage is disregarded). Thus, we used this null model as the base upon which we built 15 alternative models with various combinations of variables for each physiological state of *C. stellifer*.

The best model for overall female *C. stellifer* count featured a highly significant (α ≤ 0.05) negative coefficient for the distance to supplementary feeders variable, showing that closer proximity was correlated with a higher midge abundance. Positive coefficients suggested that adult female *C. stellifer* midges were generally in higher abundance in mixed bottomland hardwood habitats (*p* = 0.0068), then rural/developed/other habitats (*p* = 0.0423). Parous *C. stellifer* abundance was significantly higher at closer distances to supplementary feeders (*p* = 4.59 × 10^−16^) and permanent water sources (*p* = 0.0018) with habitat type excluded from the best model. Gravid midges were in greater abundance in mixed bottomland hardwood forests (*p* = 3.66 × 10^−24^) and avoided rural/developed/other habitats (*p* = 0.0003). The best-performing model for bloodfed female *C. stellifer* abundance included closer distances to feeders as a significant variable (*p* = 1.38 × 10^−6^) but excluded habitat type. Nulliparous midges selected rural/developed/other land classes (*p* = 0.0535) while avoiding mixed hardwood pine forests (*p* = 0.0078). The coefficient for mixed upland pine forests was not explicitly stated in the model summaries but instead included in the intercept. In the analyses of each physiological stage, various models competed with the best one but were eliminated on the basis of parsimony. We report the summaries of the best model for each stage in Table 4.

### 3.2. Spatial Predictions

**Figure 3:**
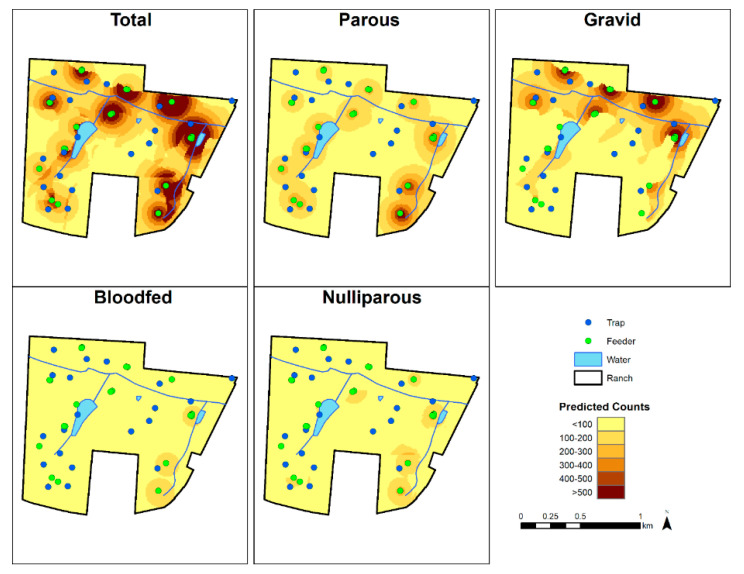
Predicted *C. stellifer* abundance on the study ranch during the seventh week of the 2016 HD transmission season (13 June to 19 June).

**Figure 4:**
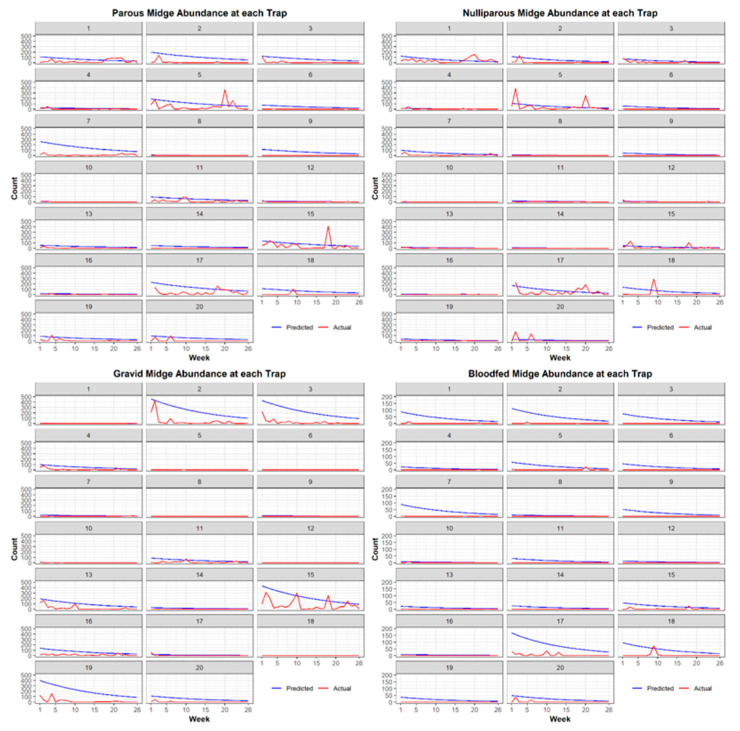
For each physiological status, predicted midge counts versus actual midge counts observed during the 2016 HD transmission season by week and trap.

As including all 26 maps for each physiological status was unfeasible, we mapped the seventh week of the 2016 HD season (13 June 2016 to 19 June 2016) to show the abundance of *C. stellifer* of each status on the study ranch (Figure 3) and as a comparison to any other week in that season. We found that all the models predicted an overall decline in midge abundance as the HD season progressed, with rapid decrease early in the transmission period (Figure 4). Each map generally agreed that two feeders in the northeastern corner of the preserve had high *C. stellifer* abundances in their vicinities (Figure 3). The map of total abundance showed relatively high predicted counts around feeders in mixed bottomland hardwood habitat. Our prediction of gravid midge counts showed that they were abundant in mixed bottomland hardwood environments, but they especially preferred areas near feeders bordering the major creek crossing the private property east-to-west. Parous midges were abundant around every feeder on the study ranch.

Since parous and gravid midges were the most and second-most likely of the physiological stages, respectively, to be infectious with EHDV and BTV, we illustrated their predicted seasonal abundance by week as animated GIFs in Figures S1 and S2 (Supplementary Material).

## 4. Discussion

Here, we applied an occupancy modeling approach to predict the distribution of *C. stellifer*, a suspected vector of EHDV and BTV in Florida. Our goal was to determine which combinations of habitat data and host density predict abundance during the EHDV transmission period. Finding that UD was not a significant factor in any of our models meant that merely having deer (possible bloodmeal hosts) available did not influence estimates of *C. stellifer* distribution and abundance. Rather, our modeling results support our first hypothesis that female *C. stellifer* of all physiological statuses, except parous, prefer different habitats and environmental resources. When modeling total midge count irrespective of physiological status, our results suggest that female *C. stellifer* preferred habitats near large-animal supplementary protein feeders in mixed bottomland hardwood habitats during the hemorrhagic disease transmission risk period.

Supplemental feeding has been studied in relation to bovine tuberculosis (TB) and brucellosis, two important bacterial pathogens affecting WTD in Michigan [34] and elk in the Greater Yellowstone Ecosystem [35]. Although bovine TB and brucellosis are typically indirectly transmitted between hosts through their contact with environmental agents contaminated by infectious bodily fluids, the idea that resource supplementation of wildlife may increase vector-borne disease transmission may also be true. For example, the American crow (*Corvus brachyrhynchos*) is an amplifying host of West Nile virus (WNV) in the US. Yaremych et al. [36] determined crows in Illinois tended to select low-to medium-density urban land cover over more forested areas, likely because the urban environment provided food sources that supplemented agricultural foraging. Consequently, this preference may expose crows to *Culex pipiens*, a synanthropic mosquito and the primary vector species of WNV in Illinois. These studies and our own data suggest that feeding areas that concentrate animal activity can potentially increase contact opportunities between hosts and vectors and may consequently act as hotspots for vector-borne disease transmission [37,38]. Additionally, increased vector–host interaction may result in more feedings by infected vectors within a single gonotrophic cycle. If multiple hosts are aggregated around a feeder and an infectious midge is interrupted while feeding on one host, the midge may move between hosts to acquire a full blood meal, potentially enhancing pathogen transmission to multiple hosts concurrently at the same feeder; while data are limited, multiple host feeding has been confirmed [39].

Our finding that female *C. stellifer* midges overall preferred large-animal supplementary protein feeders in mixed bottomland hardwood habitats did not apply to some physiological statuses. We found that midges in different physiological states preferred different resources across the landscape. For example, habitat type was not included in our best models of parous and bloodfed midge abundance and distribution, but the opposite was true for gravid and nulliparous midges. Parous midges favored sites close to feeders and permanent water bodies without selecting any particular habitat class. This can be explained by their need for blood meal hosts following oviposition in muddy environments along water margins. Gravid and bloodfed female abundance near feeders can be explained by high host density and their need to find a place to safely digest their most recent blood meal and develop and lay their eggs. Like parous midges, nulliparous abundance near feeders can be explained by their desire for blood meal hosts. Their selection of rural/developed/other land covers and avoidance of mixed hardwood pine forests may be due to their recent emergence as adults from their larval habitat. Gravid midges exhibited the opposite behaviors in regard to habitat type, likely because they are seeking suitable oviposition sites in shady mixed bottomland hardwood forests with moist soil. Since each status was best modeled with different variables, we cannot directly compare predictions and model AIC between *C. stellifer* at different physiological stages. Furthermore, the best model for each physiological stage predicted a rapid and early decline in midge abundance as the HD transmission season advanced, but our models could not capture the variability we observed in our actual midge count data. We need more time-specific variables to model this variation or to better understand how seasonal changes relate to these decreased populations late in the sampling period. Nonetheless, our study is the first published assessment of the habitat preferences of female *C. stellifer*, a candidate vector of EHDV and BTV in Florida [40] with a poorly known ecology, at various physiological states. The decline in this species ahead of the HD peak suggests that more than one species drives HD outbreaks in this study area.

Since parous female *C. stellifer* are the most likely physiological state to be infectious vectors of EHDV and BTV and favor different environments relative to their nonparous cohorts, HD risk to deer is nonrandom across the study landscape. Consequently, effective vector management for reducing HD burden should be aimed at sites near large-animal feeders and permanent water bodies, though data on the practicality and effectiveness of such measures are lacking and such control may be unrealistic [7]. For example, the high degree of site fidelity exhibited by WTD in our study site [41] could be utilized to geographically and temporally limit disease transmission [37] or focus pest management. If supplemental feeding of large animals increases disease transmission risk, discontinuing use of supplemental feeders located within high-risk habitats such as mixed bottomland hardwood forests on the study ranch may be one option to mitigate disease transmission, though we suggest that it should be done gradually to be successful. Vanderhoof and Jacobson [42] reported that although agronomic plantings were highly attractive to WTD, the animals did not drastically and immediately change their home ranges in response to newly planted supplemental food plots [43]. Instead, if supplementary food resources were placed within a deer’s home range, the animal would likely shift its core area of activity closer to the new feeding site [37]. Likewise, when deer access to feeders shifts spatially, the animals will most likely adjust their foraging behavior to acquiring natural and/or alternatively placed diets [44]. Slowly discontinuing use of feeders in mixed bottomland hardwood forests while increasing feed left in other environments may help decrease contact between parous midges and susceptible WTD. However, deer ranch managers may be hesitant or unable to stop using certain supplementary feeders on their properties, particularly when animal densities are high, so daily and seasonal timing of animal feeding may be an alternative preventative strategy against orbiviral transmission. WTD and *C. stellifer* have crepuscular feeding activity [45,46], so providing supplementary feed to deer only at daytime via automated feeders, may help reduce individual animal contact with infectious vectors during *C. stellifer* peak activity time. During winter and early spring, when natural forage may be limited and midges are not as abundant, increasing supplemental feeding may be less risky to the deer.

## Figures and Tables

**Table 1:** Covariates used to model *Culicoides stellifer* abundance in this study.

Variable	Description
Feeder	Euclidian distance from each feeder
Water	Euclidian distance from major water bodies
Habitat	Discrete habitat category: upland pine, mixed hardwood pine, mixed bottomland hardwood, or rural/developed/other
Utilization distribution (UD)	Weekly probability density of deer presence
Latitude	Insect trap location latitude
Longitude	Insect trap location longitude
Week	Week in which samples were collected

**Table 2:** Pearson correlation *r* values for testing correlation between continuous numerical variables.

	Latitude	Longitude	Feeder	Water	Weekly UD
Latitude	1.0000	0.2570	0.5661	−0.0282	−0.0789
Longitude	0.2570	1.0000	0.2170	−0.5426	−0.0789
Feeder	0.5661	0.2170	1.0000	0.3105	−0.0451
Water	−0.0282	−0.5426	0.3105	1.0000	−0.0331
Weekly UD	−0.0789	−0.2556	−0.0451	−0.0331	1.0000

**Table 3:** ANOVA *p*-values for testing correlation between continuous numerical and categorical variables.

Variables	*p*
Feeder and habitat	<2.0 × 10^−16^
Water and habitat	<2.0 × 10^−16^
UD and habitat	0.0002

**Table 4:** Best *N*-mixture models of abundance for total and each physiological status of *Culicoides stellifer* on the panhandle Florida study deer ranch during the 2016 HD season.

Status	Variable	Estimate	SE	*p*-Value
All	Intercept	4.8848	0.2365	8.11 × 10^−95^
	Week	−0.0569	0.0090	3.00 × 10^−10^
	Latitude	0.3853	0.1088	3.99 × 10^−4^
	Longitude	−0.0207	0.0910	0.8200
	Mixed hardwood pine	−0.5807	0.3770	0.1230
	Mixed bottomland hardwood	0.6705	0.2476	0.0068
	Rural/developed/other	0.4843	0.2452	0.0482
	Feeder	−0.3005	0.0962	5.29 × 10^−25^
Parous	Intercept	4.2716	0.1666	6.37 × 10^−145^
	Week	−0.0507	0.0106	1.72 × 10^−6^
	Latitude	0.1608	0.1095	0.1420
	Longitude	−0.3355	0.1046	0.0013
	Feeder	−0.9647	0.1188	4.59 × 10^−16^
	Water	−0.3005	0.0962	0.0018
Gravid	Intercept	2.5957	0.2455	3.94 × 10^−26^
	Week	−0.0613	0.0095	9.62 × 10^−11^
	Latitude	0.2636	0.0997	0.0082
	Longitude	0.3327	0.1067	0.0018
	Mixed hardwood pine	0.3859	0.4426	0.3830
	Mixed bottomland hardwood	2.5626	0.2541	3.66 × 10^−24^
	Rural/developed/other	−1.0642	0.2944	0.0003
	Feeder	−0.8350	0.10922	2.08 × 10^−14^
Bloodfed	Intercept	3.7417	0.3205	1.76 × 10^−31^
	Week	−0.0721	0.0191	0.0002
	Latitude	0.4233	0.1777	0.0172
	Longitude	−0.3422	0.1377	0.0130
	Feeder	−0.8664	0.1795	1.38 × 10^−6^
Nulliparous	Intercept	3.7178	0.2757	1.93 × 10^−41^
	Week	−0.0714	0.0108	3.69 × 10^−11^
	Latitude	0.5067	0.1268	6.43 × 10^−5^
	Longitude	−0.1940	0.1072	0.0703
	Mixed hardwood pine	−1.1957	0.4497	0.0078
	Mixed bottomland hardwood	−0.2292	0.2918	0.4320
	Rural/developed/other	0.5516	0.2857	0.0535
	Feeder	−1.0703	0.1176	9.08 × 10^−20^

## Data Availability

The datasets generated and/or analyzed during the current study are not publicly available due private landowner privacy agreement but are available from the corresponding author on reasonable request.

## Supplementary Materials

The following are available online at https://www.mdpi.com/article/10.3390/v13071328/s1, Figure S1: A GIF animation of seasonal abundance estimates by week for parous life stage *Culicoides stellifer*, a candidate vector for EHDV and BTV, at a wildlife farm in Northern Florida, Figure S2: A GIF animation of seasonal abundance estimates by week for gravid life stage *Culicoides stellifer*, a candidate vector for EHDV and BTV, at a wildlife farm in Northern Florida.
