# Supplementary figures and images for: Modeling Abundance of Culicoides stellifer, a Candidate Orbivirus Vector, Indicates Nonrandom Hemorrhagic Disease Risk for White-Tailed Deer (Odocoileus virginianus)
Source: Viruses. 2021 Jul 9;13(7):1328. doi: 10.3390/v13071328 (PMC8310359; doi:10.3390/v13071328)

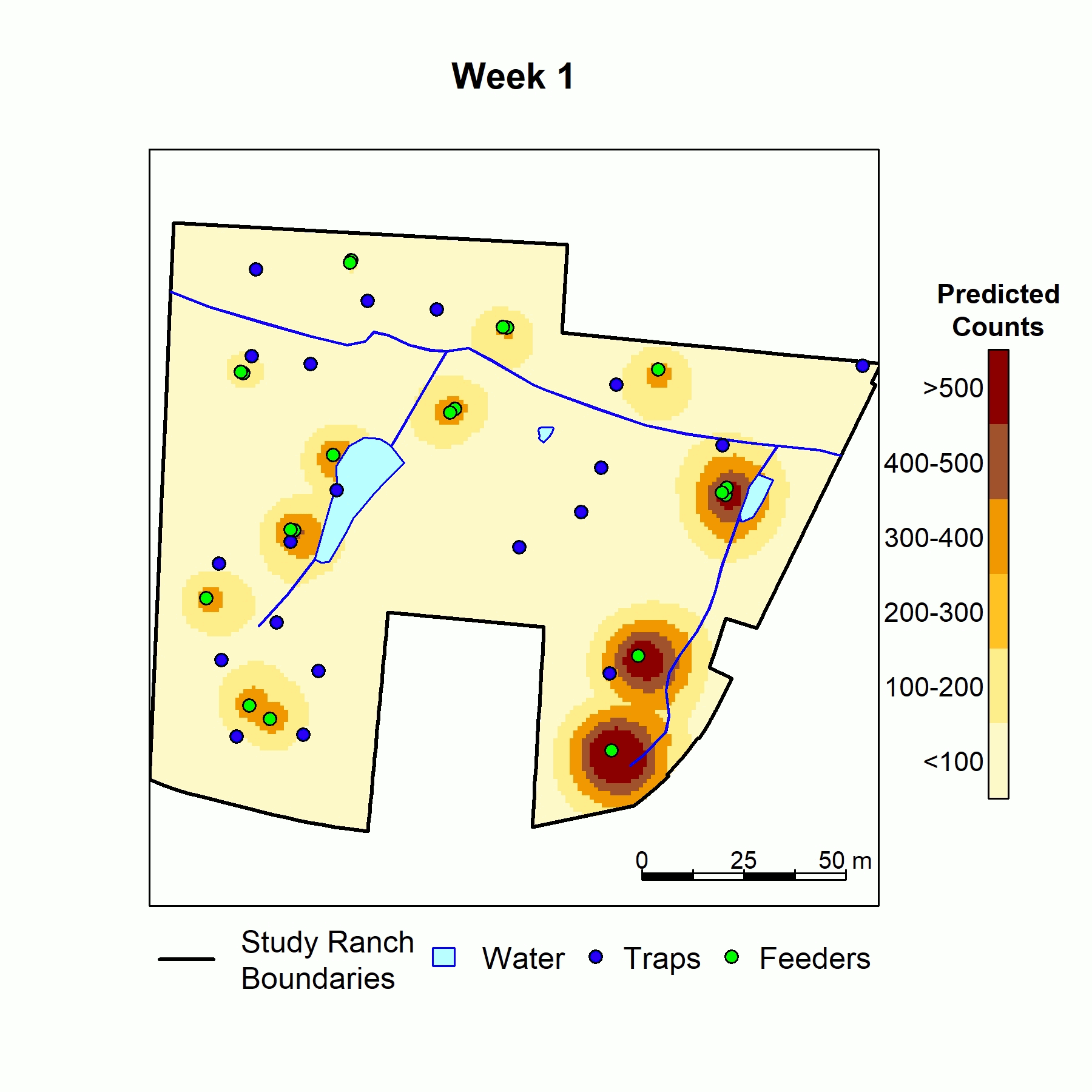

Supplement: Supplementary file 1 [file viruses-13-01328-s001.zip › FigureS1GIF.gif]

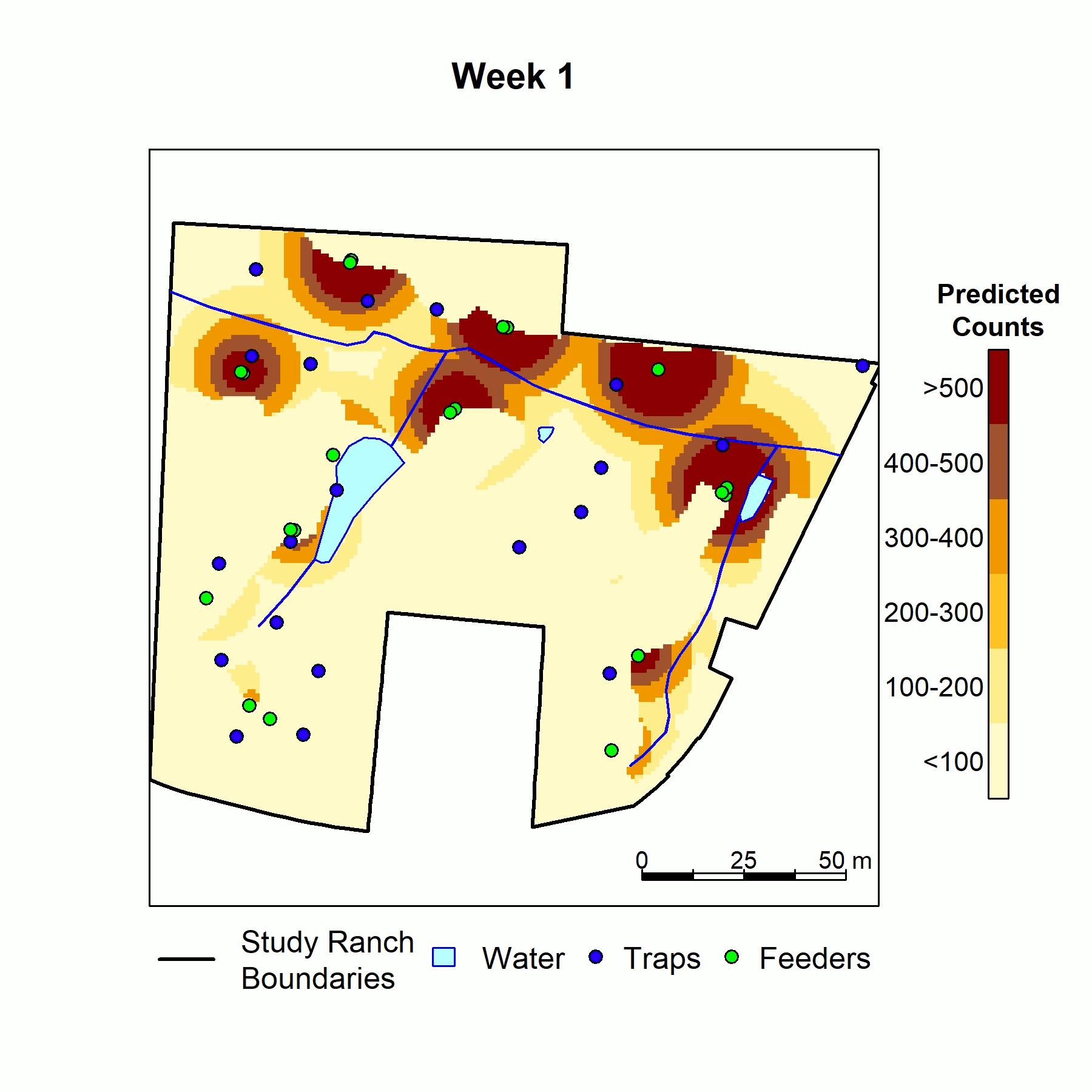

Supplement: Supplementary file 1 [file viruses-13-01328-s001.zip › FigureS2GIF.gif]
